# Culinary medicine as a new paradigm integrating clinical nutrition and medical practice: a narrative review

**DOI:** 10.3389/fnut.2026.1963264

**Published:** 2026-10-15

**Authors:** Hyung Gyu Park, Hyunjung Lim

**Affiliations:** 1 Department of Medical Nutrition, Graduate School of East-West Medical Science, Kyung Hee University, Yongin, Republic of Korea; 2 Research Institute of Medical Nutrition, Kyung Hee University, Seoul, Republic of Korea

**Keywords:** clinical nutrition, culinary medicine, dietary behavior, Food-is-Medicine, medical nutrition therapy, precision nutrition

## Abstract

Culinary medicine is an emerging interdisciplinary field that integrates nutrition science, culinary practice, behavioral support, and medical care to translate dietary recommendations into feasible meals and sustainable eating behaviors. This narrative review examines its conceptual scope, plausible mechanisms, clinical and educational applications, implementation models, and future research priorities. Culinary medicine differs from general culinary education by linking food preparation skills to evidence-based nutritional and clinical goals. It complements medical nutrition therapy, precision nutrition, and Food-is-Medicine interventions by providing the practical competencies required to select, plan, prepare, and consume meals consistent with individualized recommendations. Its potential effects operate through interrelated behavioral and physiological pathways. Hands-on culinary education may strengthen cooking self-efficacy, food skills, dietary adherence, and nutrition-related self-management. Cooking methods and food combinations may also modify protein and starch digestibility, advanced glycation end-product formation, nutrient bioavailability, postprandial metabolic responses, and microbial fermentation. Current applications include health professional education, interprofessional training, teaching kitchens, disease-specific nutrition programs, community-based interventions, and virtual or hybrid delivery. Existing evidence more consistently supports improvements in cooking confidence, self-efficacy, dietary behavior, and self-management than sustained changes in body weight, glycemic control, blood pressure, lipid profiles, or disease incidence. Interpretation is limited by small samples, short follow-up periods, heterogeneous intervention components, non-randomized designs, and reliance on self-reported outcomes. Conceptual imprecision, resource requirements, and inequities in time, food access, kitchen facilities, and financial capacity further constrain implementation. Future studies should evaluate culinary medicine as an implementation strategy embedded within nutrition care rather than as a stand-alone cooking program. Programs should define core components and professional roles, adapt delivery to cultural and socioeconomic contexts, and assess adherence, participant burden, cost, healthcare utilization, and long-term clinical outcomes. Culinary medicine may thereby serve as a practical bridge between nutritional science, individualized nutrition care, and everyday meal preparation.

## 1 Introduction

Unhealthy diet and nutrition are increasingly recognized as critical determinants of health maintenance and chronic disease prevention, prompting substantial interest in integrating nutrition into clinical care (1–3). Nevertheless, a persistent gap remains between nutritional knowledge and its application in real-world practice. Although the evidence base linking dietary patterns to health outcomes has grown considerably, practical methods for translating this evidence into sustainable changes in food choices and eating behavior remain underdeveloped (4, 5). This gap has been closely associated with the long-standing observation that nutrition is inadequately addressed in medical education, which has served as one factor for the emergence of culinary medicine (CM) (6, 7).

CM integrates culinary practice, nutrition science, and medicine, extending beyond the transfer of knowledge toward an experiential, skills-based approach through which individuals learn to plan, prepare, and sustain healthful meals (8, 9). In this respect, it aligns with the broader “Food-is-Medicine (FIM)” paradigm, which seeks to embed food and nutrition within the healthcare system, and it is characterized by an emphasis on operationalizing nutrition as practicable and sustainable behavior rather than abstract recommendation (5, 10).

CM holds particular relevance at its interface with clinical nutrition and precision nutrition. Whereas clinical nutrition manages the nutritional status of each patient through medical nutrition therapy, CM translates these principles into the applied domain of food and cooking, facilitating their joint application by clinicians and patients (9, 11). Moreover, the goal of precision nutrition can be realized in a practicable form only when evidence-based dietary guidance is implemented at the level of the meal, a translation that CM is well positioned to enable.

This narrative review examines CM as an emerging paradigm integrating clinical nutrition with medical practice. Specifically, we delineate the definition and conceptual scope of CM, examine the mechanisms through which it may contribute to disease prevention and management, and discuss its application and implementation, together with current challenges and future directions.

## 2 Methods

### 2.1 Study design

This study was conducted as a structured narrative review to synthesize the conceptual foundations, plausible mechanisms, clinical and educational applications, and implementation considerations of CM. Consistent with methodological recommendations for narrative reviews, the review process emphasized a clearly defined objective, transparent literature identification, appropriate use of evidence, and scientifically reasoned synthesis (12). To address the main purpose of this literature review, a comprehensive conceptual domain was defined in advance, and the specific details within each domain were refined during the descriptive synthesis of the literature.

### 2.2 Literature search strategy

Relevant literature was identified through searches of PubMed/MEDLINE, Embase, and Web of Science from database inception through August 2026. The primary search focused on CM using combinations of terms including “culinary medicine,” “culinary nutrition,” “teaching kitchen,” “cooking intervention,” and “cooking education.” Because CM encompasses behavioral, nutritional, biochemical, and clinical domains, supplementary targeted searches were conducted for topics relevant to the conceptual framework of this review, including cooking self-efficacy and skills, cooking methods and food processing, advanced glycation end products, starch digestibility and resistant starch, nutrient bioavailability, postprandial metabolic responses, medical nutrition therapy, FIM, precision nutrition, and clinical or educational applications of culinary interventions. Reference lists of relevant reviews and key articles were also examined to identify additional publications.

### 2.3 Literature selection and synthesis

Peer-reviewed original studies, systematic reviews and meta-analyses, relevant narrative reviews, consensus or professional guidance, and seminal conceptual articles were considered when they directly informed the predefined domains of this review. Human studies and higher-level evidence, including systematic reviews, randomized or controlled interventions, and prospective studies, were preferentially used for clinical and behavioral claims. Experimental or food-science studies were additionally considered when necessary to explain biological or food-processing mechanisms.

The literature was organized and synthesized using predefined conceptual domains from the author. The major domains were established *a priori* from the objectives of the review and an initial appraisal of the scope of the CM literature: (1) the definition and conceptual positioning of CM; (2) plausible behavioral, physiological, and nutritional mechanisms; (3) clinical, educational, and implementation applications; and (4) current limitations and future research directions. These domains were selected to provide a progression from defining what CM is and how it differs from adjacent approaches, through the mechanisms by which it may operate, to its application in practice and the limitations relevant to its future development. Within these broad domains, the organization of specific subtopics was refined iteratively during literature synthesis to reflect concepts consistently relevant to the evidence reviewed. Evidence was integrated narratively within each domain rather than quantitatively; therefore, no formal meta-analysis, qualitative thematic analysis, or systematic risk-of-bias assessment was performed.

## 3 Understanding culinary medicine

### 3.1 Definition and conceptual scope

CM has been defined as an evidence-based field that combines the art of food and cooking with the science of medicine (8). Recent definitions characterize it as a multidisciplinary approach that integrating nutrition knowledge, food preparation skills, and behavioral support to enable individuals to make health-promoting food choices across clinical, educational, and community settings (13, 14).

The development of CM has been closely associated with efforts to strengthen nutrition education within medical and other health-professional training (6, 7, 15). In response to persistent gaps in nutrition competency among healthcare professionals, medical schools and teaching-kitchen programs have incorporated experiential culinary education alongside nutrition science, clinical case discussion, and dietary counseling (6, 15, 16).

The scope of CM has subsequently expanded beyond health-professional education to patient care and community-based health promotion (14, 17). Taken together, these developments support conceptualizing contemporary CM as a translational approach rather than a single standardized intervention, with applications spanning professional education, prevention, chronic disease management, and support for the implementation of individualized dietary recommendations (14, 15, 17). Across these settings, its defining focus is the translation of evidence-based nutrition principles into practicable food- and meal-level behaviors that can be adapted to clinical goals and real-world circumstances (8, 9, 18).

### 3.2 The distinguishing emphasis on application

The distinguishing feature of CM is its focus on application (9, 19). Whereas conventional nutrition education primarily conveys recommendations regarding foods, nutrients, or dietary patterns. CM further teaches individuals how to translate these recommendations into feasible meals. Typical components include hands-on food preparation, recipe modification, meal planning, food-label interpretation, grocery shopping, portion management, cooking-method selection, and sensory evaluation, as well as strategies for using leftovers or available ingredients (15, 19). Programs may additionally incorporate goal setting, self-monitoring, problem-solving, and clinical case discussion (20).

### 3.3 Interprofessional collaboration and delivery

CM is inherently interdisciplinary. Registered dietitian nutritionists contribute expertise in nutritional assessment and disease-specific dietary management. Physicians and other healthcare professionals connect food practices with clinical conditions and treatment plans. Culinary professionals translate nutritional targets into acceptable recipes and cooking procedures. Community organizations may provide food resources and social support (18).

Delivery settings are correspondingly diverse, encompassing teaching kitchens, healthcare facilities, community centers, schools, workplaces, grocery stores, or digital platforms (19). Target populations range from healthcare trainees and healthy adults to individuals with obesity, diabetes, cardiovascular risk factors, or other diet-related conditions (15). This breadth affords considerable translational potential but also contribute to substantial heterogeneity in intervention content and outcomes (20).

### 3.4 Differentiation from adjacent concepts

CM can be differentiated from general culinary education by its purpose, mode of delivery, and target outcomes. General culinary education focuses principally on developing food and cooking skills and does not necessarily link these competencies to evidence-based clinical objectives (21). In contrast, CM integrates nutrition education with experiential food preparation to help individuals apply health-related dietary recommendations in everyday settings. A defining characteristic of a CM intervention is that it is delivered by healthcare professionals, such as physicians, nurses, or registered dietitians, rather than by chefs or culinary experts alone (22). CM further occupies a distinct yet complementary position relative to medical nutrition therapy (MNT) and FIM interventions. MNT constitutes individualized, disease-specific nutrition care provided by qualified nutrition professionals through nutrition assessment, diagnosis, intervention, and monitoring and evaluation (23, 24). FIM interventions primarily improve access to health-promoting foods through healthcare-linked services, including medically tailored meals, medically tailored groceries, and produce prescriptions (5, 25). Within this framework, CM complements these approaches by developing the practical competencies required to select, prepare, and incorporate foods into meals consistent with nutritional and clinical goals. Consequently, it may contribute to individuals implementing recommendations arising from MNT and effectively using foods supplied through FIM programs. Figure 1 suggests the conceptual positioning of CM as a translational discipline linking general culinary education, clinical nutrition, precision nutrition, and FIM interventions based on the literature reviewed. General culinary education provides foundational food-preparation skills, MNT defines individualized therapeutic nutrition goals, FIM facilitates access to health-promoting foods and food-related resources, and precision nutrition informs the individualization of dietary strategies. CM may be positioned as the practical interface through which these inputs can be translated into feasible behaviors.

**FIGURE 1:**
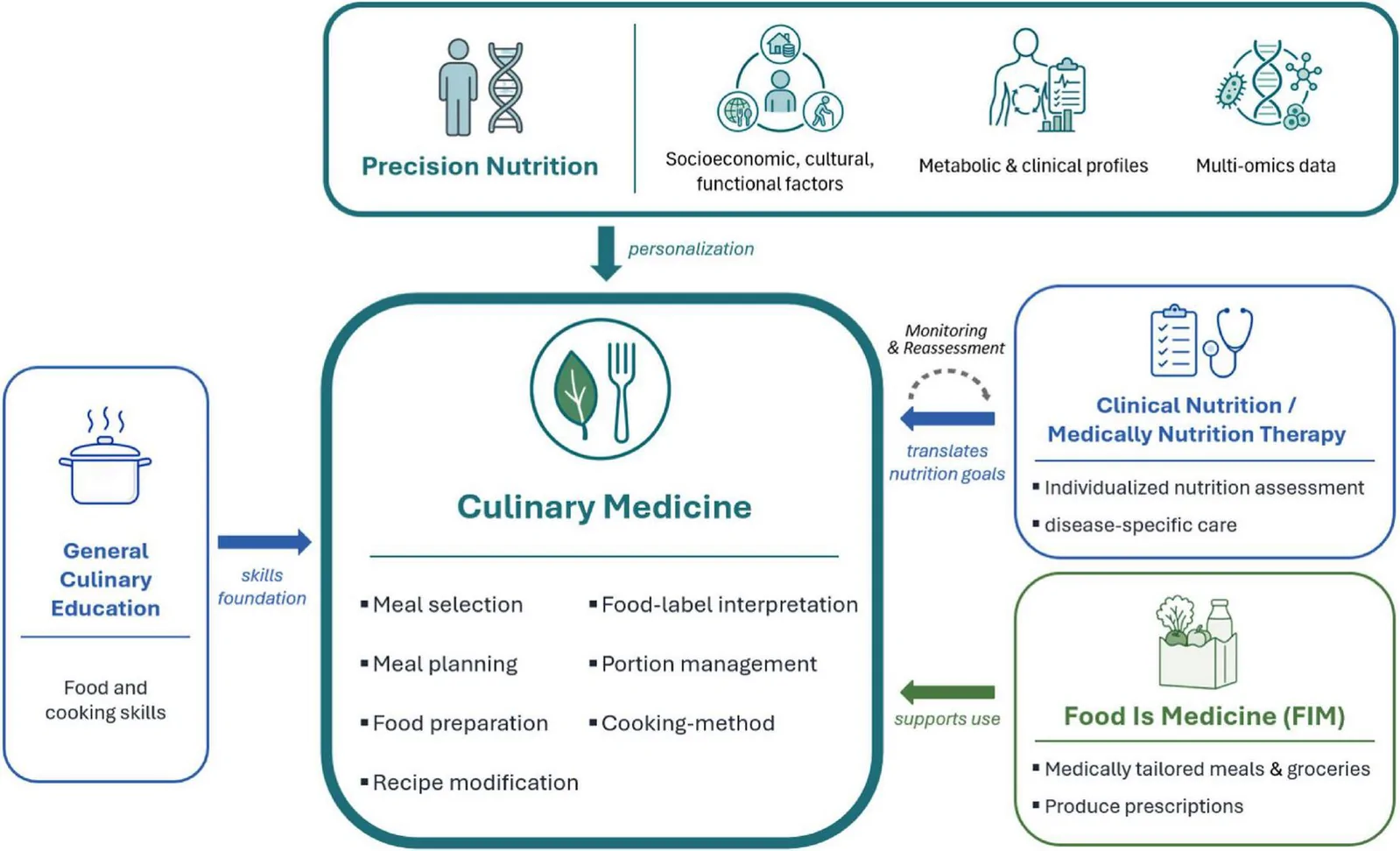
Conceptual framework of culinary medicine as a translational approach to applied nutrition. The bidirectional relationship between CM and MNT reflects an iterative process in which clinical nutrition goals inform culinary strategies, while monitoring of adherence, feasibility, and dietary response may subsequently inform reassessment and modification of nutrition care.

## 4 Plausible mechanisms for disease prevention and management

The potential health effects of CM can be conceptualized through several behavioral and biological pathways. Culinary education may first improve nutrition knowledge, cooking skills, and cooking-related self-efficacy (26, 27), which in turn promote more frequent and health-promoting meal preparation, healthier food selection, and sustained dietary practices (28). These behavioral changes translate into altered dietary exposure, which may subsequently influence body-weight regulation, glycemic control, lipid metabolism, inflammatory status, and gut microbial function (29, 30). Beyond food selection, culinary technique itself modifies the biochemical properties of what is consumed, and the manner in which foods are combined further shapes nutrient bioavailability and postprandial metabolic responses (31, 32). Collectively, these findings suggest that behavioral and biological pathways may interact, providing a rationale for considering their individual variability within a precision nutrition framework.

### 4.1 Changing cooking behaviors and enhancing self-efficacy

The behavioral mechanism of CM is most coherently framed within social cognitive theory, in which self-efficacy is a principal determinant of behavior change and is acquired chiefly through mastery experience (20, 33). Hands-on culinary education is structured to generate precisely such experiences, e.g., participants observe a technique, perform it under guidance, receive immediate feedback, and consume the resulting meal (19). Repeated successful preparation of simple meals thereby reduces perceived difficulty and builds confidence in handling unfamiliar foods, modifying recipes, and preparing meals without strict reliance on written instructions (34, 35).

This mechanism is particularly relevant when dietary prescriptions are complex and require individuals to make ongoing decisions regarding food selection, preparation, timing, and portioning rather than simply adhere to fixed dietary rules (36–38). CM allows participants to rehearse these disease-specific tasks rather than to receive information alone (27). Structured meal templates, ingredient-substitution guides, and standardized cooking ratios further reduce cognitive demand (39). Teaching a flexible framework such as a whole grain, a protein source, vegetables, and a minimally processed sauce is more transferable than a single fixed recipe (35) and can be adapted to available foods, cultural preferences, and dietary restrictions (40).

Systematic review evidence indicates that culinary interventions improve attitudes, self-efficacy, cooking confidence, and dietary intake more consistently than body weight, blood pressure, or blood lipids (19). Therefore, cooking competence should be regarded not as a surrogate for clinical benefit but as a mediator through which longer-term benefit may plausibly be realized (34).

### 4.2 Physiological and biochemical mediating pathways

Culinary skills such as cooking methods may role-mediate influence biological exposure. Moderate heating can unfold protein structures and expose peptide bonds to digestive enzymes, thereby improving digestibility in some foods (41, 42). In contrast, high-temperature dry-heat techniques, including prolonged grilling, roasting, baking, and frying, can promote Maillard reactions, lipid oxidation, and the formation of advanced glycation end products (AGEs) (43, 44). Moist-heat methods, such as boiling, steaming, stewing, and poaching, generally generate fewer dietary AGEs under comparable conditions (45). A recent randomized crossover study found that lower-AGEs cooking methods, including boiling and steaming, reduced circulating AGEs measures and improved the lipid profile compared with higher-AGE methods (46). In addition, intense dry-heat cooking of animal protein can generate heterocyclic aromatic amines (HAAs) through reactions involving amino acids (47), the formation of which is influenced by cooking temperature, duration, and heat-transfer method (48). In prospective U.S. cohorts, frequent open-flame and/or high-temperature cooking of meat was associated with a higher risk of incident type 2 diabetes (T2D) independent of total meat intake and baseline BMI, and higher estimated intake of HAAs was also positively associated with diabetes risk (49). These results make it impossible to establish a causal relationship or determine whether the compounds generated during the cooking process are the cause of the increased risk, as residual confounding variables related to dietary and lifestyle factors remain. However, these results provide a mechanistic rationale for evaluating whether CM interventions that modify cooking practices can meaningfully alter such dietary exposures.

Starch digestibility is another potential pathway. Cooking disrupts starch granules and promotes gelatinization, generally increasing enzymatic accessibility and postprandial glucose availability (50, 51). However, during subsequent cooling, gelatinized starch chains can reassociate through retrogradation, generating resistant starch that is less susceptible to digestion in the small intestine (52). Because resistant starch escapes substantial digestion in the small intestine, it can reach the colon and provide a fermentable substrate for the gut microbiota (53). Its microbial degradation produces short-chain fatty acids, particularly acetate, propionate, and butyrate, which contribute to colonocyte energy metabolism, intestinal barrier integrity, immune regulation, and host metabolic signaling (53, 54). Consistent with this, one clinical study reported that cooked white rice cooled under refrigeration and subsequently reheated produced a lower glycemic response than freshly cooked rice (55).

### 4.3 Food and meal combinations

CM may support disease prevention and management by optimizing not only individual food choices but also manner in which foods are combined within meals, as food combinations can influence nutrient bioavailability and postprandial metabolic responses (56–58).

A representative example is the combination of tomatoes and olive oil. Lycopene is a lipophilic carotenoid, and its absorption can be enhanced by thermal processing and the presence of dietary fat. In a human intervention study, consumption of tomatoes cooked with olive oil significantly increased plasma lycopene concentrations compared with tomatoes cooked without olive oil (59). Similarly, adding avocado or avocado oil to vegetable-based meals increased the absorption of carotenoids, including lycopene, β-carotene, and lutein (57). These findings suggest that pairing carotenoid-rich vegetables with healthy fat sources may improve the bioavailability of fat-soluble phytochemicals.

Food combinations may affect mineral absorption. Non-heme iron, which is found in plant foods such as legumes, grains, and leafy vegetables, is strongly influenced by meal composition (58). Ascorbic acid enhances non-heme iron absorption by reducing ferric iron to ferrous iron and forming soluble iron complexes in the intestinal lumen (60, 61). From a culinary perspective, this supports practical pairings such as legumes with tomatoes, leafy greens with citrus juice, or iron-rich plant foods with bell peppers. However, the effect may vary depending on the presence of inhibitors such as phytate and polyphenols, as well as individual iron status. Citrus juice or ascorbic acid may improve the digestive recovery of green tea catechins (62).

Meal combinations can also influence postprandial glucose metabolism. Vinegar or acetic acid consumed with carbohydrate-rich meals has been shown to attenuate postprandial glucose and insulin responses in clinical trials and meta-analyses (56).

### 4.4 Individualization through precision nutrition

A recurrent observation in clinical nutrition is that dietary protocols effective in one cohort may yield different outcomes in another, reflecting systematic inter-individual variation in the response to identical foods (63–68).

For example, an individual exhibiting exaggerated postprandial glycemic responses may benefit not only from a lower carbohydrate target but also from adjustments in carbohydrate portion size, selection of less rapidly digestible carbohydrate sources, meal composition, and preparation practices that modify starch digestibility. Similarly, individualized sodium or protein intake strategy targets can be operationalized through ingredient substitution, portion adjustment, recipe modification, and cooking techniques that maintain palatability or accommodate functional limitations (e.g., anorexia, dysphagia, difficulty chewing). Within the conceptual framework proposed in this review, precision nutrition determines what should be modified, whereas CM provides practical strategies for how those modifications can be implemented at the meal level.

However, much of the evidence supporting these physiological and biochemical pathways derives from studies of specific cooking methods, food preparation practices, or meal combinations rather than from CM interventions themselves. Accordingly, these findings are not interpreted as direct evidence of CM-mediated clinical benefit. Nevertheless, future research and CM program planning should account for these mechanistic factors by adopting a precision nutrition approach that comprehensively considers not only participants’ characteristics, including socioeconomic, cultural, and functional aspects but also their metabolic profiles and omics data.

## 5 Application and implementation of culinary medicine

CM is not a single intervention format but has been adapted according to target population, clinical purpose, delivery setting, and available resources. Its utility is best assessed by the functions it performs within existing structures of care.

### 5.1 Educational applications

Health professional education remains the most established application of CM. Curricula typically combine hands-on cooking, nutrition education, case-based discussion, and counseling practice, and have reported improvements in nutrition knowledge, cooking confidence, and perceived counseling ability, although delivery methods, duration, and content vary substantially across institutions (6, 13, 15).

CM has also been incorporated into interprofessional education involving medical, nursing, and dietetics students. The aim is to establish a shared vocabulary and baseline competence sufficient for clinicians to recognize dietary contributors to disease, initiate credible dietary conversations, and refer appropriately to nutrition professionals and food-based programs (69–71).

Beyond health professional education, CM has increasingly been applied as a patient- and community-facing educational strategy across diverse populations and delivery settings. Teaching-kitchen, group-based, and virtual programs have been implemented among individuals with diabetes (27, 72–76), obesity or cardiometabolic risk (77–80), hypertension (81), and cancer-related nutritional challenges (82), as well as in community-based family populations (83). Across these interventions, improvements have been reported in cooking confidence and self-efficacy (74, 75, 77, 82), dietary self-management (27, 75), adherence to healthful dietary patterns (79, 83), food literacy (76), and dietary intake (78, 81). In contrast, changes in clinical and metabolic outcomes, including body weight, glycemic control, blood pressure, and lipid profiles, have been less consistent across studies (72–74, 78–81). Intervention studies based on CM are summarized in Table 1.

**TABLE 1:** Clinical studies evaluating culinary medicine interventions.

Study	Countries	Population	Sample size	Study design	Intervention	Comparator	Duration/ follow-up	Principal behavioral/ dietary outcomes	Clinical outcomes
Dasgupta et al. (72)	Canada	Adults with T2DM and BMI 25–40 kg/m^2^	72 enrolled; 53 completed	Single-arm phase 2 pre–post trial	Meal-preparation training by chef + nutrition/eating-behavior education by a registered dietitian	–	24 weeks; 15 × 3-h sessions	WEL +11.2 points (95% CI 4.7–17.8); steps +869/day (95% CI 198–1,540)	Weight −2.2% (95% CI −3.6 to −0.8); HbA1c −0.3% (95% CI −0.6 to −0.1)
Monlezun et al. (73)	USA	Adults with T2DM	27 randomized	Pilot randomized controlled trial	Mediterranean diet–based hands-on cooking and nutrition curriculum	MNT/standard nutrition education	6 months	Correct-portion belief +18% vs. −11% (*p* = 0.124); label use +34% vs. 0% (*p* = 0.745)	DBP −4 vs. +7 mmHg (*p* = 0.037); total cholesterol −14 vs. +17 mg/dL (*p* = 0.044); HbA1c NS (*p* = 0.575)
Eisenberg et al. (77)	USA	Adults with ≥1 cardiovascular risk factor	40	Single-arm feasibility pilot	Teaching Kitchen curriculum: culinary instruction, nutrition, exercise, mindfulness, health coaching	–	14–16 weeks; 6- and 12-month follow-up	Cooking frequency/confidence improved (descriptive; p NR)	Weight −2.8 kg; waist −2.2 in; SBP −7.7 mmHg; DBP −6.3 mmHg; total cholesterol −7.5 mg/dL (all *p* < 0.05)
Allen-Winters et al. (82)	USA	Head and neck radiotherapy patients and caregivers	7 attended ≥1 class; 4 completed all 3	Single-arm feasibility pilot	Monthly cooking classes integrating nutrition and food-skills training	–	3 months	Healthful eating score 1.5→1.7; healthier food choices/cooking confidence improved (p NR)	No formal metabolic endpoint
Razavi et al. (83)	USA	Families with ≥1 child and ≥1 parent	41 families randomized; 145 individuals	Randomized controlled trial	Teaching-kitchen culinary education focused on Mediterranean diet principles	General dietary counseling	6 weeks	Mediterranean diet adherence ↑ (*B* = 0.43, *p* < 0.001); home-prepared meals OR 1.56 (95% CI 1.08–2.25)	Clinical/metabolic biomarkers not assessed
Williams et al. (27)	USA	Adults with T1 or T2DM	48 randomized	Waitlist-controlled randomized trial	Cooking Matters for Diabetes: cooking + DSMES + weekly food provision	Waitlist control	6 weeks; 3-month follow-up	General diet self-care +1.51 (*p* = 0.015); HEI vegetable score +0.917 (*p* = 0.023); mental HRQoL +6.7 (*p* = 0.025)	HbA1c between-group change NS post-intervention (*p* = 0.501) and at 3 months (*p* = 0.84)
Baden et al. (78)	Japan	Adults with metabolic-risk phenotype	24 randomized; 20 completed	Randomized feasibility comparison	Four 2-h classes combining nutrition, exercise, mindfulness, hands-on cooking, web support, and health coaching	Weekly vs. biweekly delivery	1–2 months; observed through 3 months	Total fat ↓ (*p* = 0.03); sodium ↓ (*p* = 0.008); HRQoL domains improved (*p* ≤ 0.047)	Weight, BMI, body fat mass ↓ (all *p* < 0.001); DBP ↓ (*p* = 0.03)
Heredia et al. (75)	USA	Adults with T2DM	104 enrolled; 61 complete posttest	Mixed-methods, intervention-only pilot	Five live virtual dietitian-led CM sessions; participants cooked from home	–	5 sessions; 6-month follow-up	Knowledge, cooking self-efficacy, fruit/vegetable intake, cooking/eating behaviors, and self-management improved (all *p* < 0.05)	HbA1c ↓ post-intervention (*p* = 0.02) and at 6 months (*p* < 0.001)
Heredia et al. (74)	USA	Low-income primary-care clinics patients with T2DM and BMI > 25	89 enrolled; 67 retained	Mixed-methods quasi-experimental pilot	Five weekly virtual CM sessions with hands-on cooking, nutrition education, and ingredient support	Non-intervention clinic controls	5 weeks	Cooking self-efficacy β = 0.43 (*p* = 0.04); barriers to healthy eating β = −0.64 (*p* = 0.03)	HbA1c ↓ within intervention group; between-group difference NS
Polak et al. (79)	USA/Israel	Adults with BMI 27.5–35 kg/m^2^ and <5 home-cooked meals/week	75 enrolled; 50 modified ITT	Bi-center randomized controlled trial	Remote CM + health coaching; dietary counseling + twelve 30-min CM/coaching sessions	Dietary counseling + CM resources	12 months	Mediterranean diet score net +1.62 at 3 months (*p* = 0.020); energy intake net −390 kcal/day at 6 months (*p* = 0.015)	Net weight change −2.52% at 3 months (*p* = 0.016), −2.98% at 6 months (*p* = 0.027), −4.30% at 12 months (*p* = 0.021)
Alcorn et al. (81)	USA	Adults with hypertension	17 completed	Randomized controlled feasibility trial	Electronic CM/nutrition education using herbs/spices to support vegetable intake	Control group	6 weeks; 1-month follow-up	Sodium ↓ (*p* = 0.008); energy ↓ (*p* = 0.02); fat ↓ (*p* = 0.005); fiber ↑ within intervention (*p* = 0.01)	DBP ↓ within intervention group (*p* = 0.003)
Krenek et al. (80)	USA	Adults with ≥5% estimated ASC disease risk	40	Randomized crossover trial	Weekly virtual cooking classes with whole-food plant-based vegan diet	High-EVOO vs. low-EVOO diet	4 weeks/phase; 1-week washout	–	LDL-C, total cholesterol, ApoB, glucose, and hs-CRP ↓ vs. baseline (all *p* < 0.05)
Stroud and Sastre (76)	USA	Rural, uninsured adults with type 2 diabetes treated in safety-net clinics	150	Single-group pre–post study with process evaluation	Produce prescription + food-literacy and culinary group classes	–	20 weeks	Food literacy 89.1→100.9 (*p* < 0.001); whole grains ↑ (*p* < 0.001); fruit ↑ (*p* = 0.03); vegetables ↑ (*p* < 0.001)	No controlled clinical/metabolic outcome reported

ASCVD, atherosclerosis cardiovascular disease; ApoB, apolipoprotein B; DBP, diastolic blood pressure; CM, culinary medicine; DSMES, diabetes self-management education and support; EVOO, extra-virgin olive oil; HbA1c, glycated hemoglobin; HEI, Healthy Eating Index; HRQoL, health-related quality of life; hs-CRP, high-sensitivity C-reactive protein; MNT, medical nutrition therapy; NR, not reported; NS, not significant; SBP, systolic blood pressure; T1DM, type 1 diabetes mellitus; T2DM, type 2 diabetes mellitus; WEL, weight efficacy lifestyle.

### 5.2 Integration with clinical nutrition and FIM programs

The clinical contribution of CM is translational (8). MNT establishes what a patient must achieve through assessment, diagnosis, intervention, and monitoring (84), but does not specify how the prescription is to be enacted within the constraints of a given household, budget, and food culture. A carbohydrate target, sodium restriction, or protein requirement defines an endpoint, not a meal (11, 84). CM addresses this implementation gap, and the same logic extends to precision nutrition, whose individualized targets remain inert unless translated into food that is prepared and consumed (68).

Disease-specific interventions support this framing. In T2D, dietary self-management requires recurrent meal-level decisions regarding carbohydrate quantity and quality, meal timing, portion size, and food preparation strategies that are feasible within household resources and food-access (36, 85). Early meal-preparation training improved eating behaviors and contributed to reductions in glycemia and blood pressure among participants who persisted with the program (72). The Cooking Matters for Diabetes trial, combining hands-on cooking, diabetes self-management education, and food provision, improved diet-related self-care and quality of life, although glycemic effects were less consistent (27). In the SPICE randomized clinical trial, a behavioral intervention emphasizing herbs and spices helped participants maintain lower sodium intake after controlled feeding (37), illustrating how culinary technique renders an otherwise unpalatable prescription sustainable.

As presented within Figure 1, FIM interventions primarily reduce food-access barriers by providing or prescribing health-promoting foods, whereas CM addresses the practical skills and decision-making required to incorporate these foods into meals that are acceptable, feasible, and consistent with nutritional goals. Produce prescriptions and healthcare-linked food provision can improve access to health-promoting foods; however, access alone may not be sufficient to ensure appropriate preparation or incorporation into sustained dietary patterns (5, 86). In clinic-integrated models combining food prescriptions with CM among low-income, food-insecure adults with diabetes, participants valued practical culinary skills, and food access alone was insufficient where individuals lacked the skills and confidence to prepare foods appropriately (87). This result suggests that effective implementation depends on the ability to translate available foods into appropriate meals and dietary patterns.

### 5.3 Delivery models

Teaching kitchens provide a structured environment in which nutrition principles are learned alongside cooking technique, often combined with other lifestyle medicine components (20). A Japanese teaching kitchen pilot integrating culinary education, lifestyle modification, and web-based self-monitoring proved feasible among adults with obesity or other diet-related risk factors and showed favorable changes in several disease-risk indicators, although the study was small and uncontrolled (78).

Because physical teaching kitchens entail substantial infrastructure and personnel requirements, virtual and hybrid models have emerged. The Nourishing the Community through CM program delivered synchronous classes streamed from a teaching kitchen to ethnically diverse adults with T2D (88); a subsequent evaluation in low-income patients with T2D and elevated BMI reported feasibility, acceptability, improved cooking self-efficacy, and reduced perceived barriers to healthy eating, although between-group biometric effects were limited (74). Virtual delivery thus preserves core elements of experiential learning while lowering the threshold to participation. The conditions under which such models can be sustained and scaled are considered below.

## 6 Challenges and future directions of culinary medicine

### 6.1 Current limitations

#### 6.1.1 Limited clinical evidence

Existing studies are characterized by small samples, short follow-up, non-randomized designs, and reliance on self-reported outcomes (15, 34). Improvements in knowledge, cooking confidence, self-efficacy, and dietary behavior are reported with reasonable consistency; evidence for sustained effects on body weight, blood pressure, glycemic control, lipids, or disease incidence is markedly less so (34). Existing studies are characterized by small samples, short follow-up, non-randomized designs, and reliance on self-reported outcomes (14, 34). Although improvements in nutrition knowledge, cooking confidence, self-efficacy, and dietary behaviors have been reported relatively consistently, evidence for sustained effects on clinical outcomes such as body weight, glycemic control, blood pressure, and lipid profiles remains limited (18, 34). Thus, culinary competence should be regarded as a potential behavioral mediator rather than a surrogate for clinical benefit, and adequately powered trials with extended follow-up are needed to determine whether these behavioral improvements translate into clinically meaningful outcomes.

Selection bias also warrants consideration and has been identified as one of the major limitations in previous reviews and cooking-based intervention studies (15, 34, 89–91). Individuals who voluntarily enroll in cooking-based interventions may have greater pre-existing interest in nutrition, food preparation, or behavioral change. Such self-selection may increase engagement and adherence, potentially limiting the generalizability of findings to broader clinical populations with lower motivation or greater barriers to participation.

#### 6.1.2 Conceptual imprecision

Terms central to the field–“real food,” “home cooking”–are invoked as though inherently health-promoting. Previous observational studies have reported that more frequent home cooking or consumption of home-prepared meals is associated with better diet quality, nutrient adequacy, and, in some populations, more favorable cardiometabolic profiles (92, 93). However, some studies reported that home-prepared meals may be high in sodium, added sugars, saturated fat, or energy density (94–96). It is cooking method, ingredient selection, and nutrient composition, not home preparation *per se*, that determines biochemical exposure. These contrasting findings highlight the need to approach CM from a precision nutrition perspective, in which culinary practices are adapted not simply to increase cooking frequency but to align food selection, meal composition, and preparation methods with individual metabolic, clinical, functional, cultural, and socioeconomic characteristics.

#### 6.1.3 Resource requirements

Kitchen facilities, equipment, food procurement, trained personnel, and protected time entail costs that are not routinely reimbursed (6, 13, 18). Heterogeneity across programs in duration, intensity, personnel, and outcome measurement further impedes comparison and the establishment of implementation standards.

#### 6.1.4 Equity and burden

CM presupposes sufficient time, cooking skill, kitchen access, food literacy, and financial resources. These conditions are least reliably met among those bearing the greatest burden of diet-related disease, who may concurrently face food insecurity, unstable work schedules, limited transportation, or inadequate cooking facilities (87, 95). Recommending fresh ingredients and daily home cooking to such populations risks increasing burden rather than improving adherence.

### 6.2 Future directions

For CM to function as a practical implementation strategy for precision nutrition in real-world settings and to become effectively integrated into healthcare systems, the field should advance in four key directions.

(1) CM should be evaluated as an implementation strategy that translates MNT, precision nutrition, and FIM recommendations into practice, rather than as a stand-alone cooking program.

(2) CM programs should be adapted to participants’ cultural, economic, functional, and food-access contexts via community co-design, with delivery extending beyond home cooking to prepared foods, batch cooking, medically tailored groceries, and digital education.

(3) CM programs should adopt multidisciplinary care models: physicians and nurses identify clinical priorities and initiate referrals, dietitians set individualized MNT-based goals and monitoring, culinary professionals translate goals into feasible meals, and community welfare workers support access and retention.

(4) Future trials should evaluate outcomes across the full pathway from acquisition of culinary competencies to sustained dietary behavior and subsequent clinical benefit. In addition to cooking knowledge, skills, and self-efficacy, studies should assess whether these changes translate into improved cooking practices, diet quality, and adherence, and ultimately into clinically meaningful changes in disease-related outcomes such as glycemic control, blood pressure, lipid profiles, and body weight. Furthermore, extended follow-up will be necessary to determine whether early behavioral improvements are sustained.

Several limitations of this review should be acknowledged. As a narrative review, literature identification and selection were guided by relevance to the predefined conceptual domains rather than by a formal systematic screening process. Consequently, selection bias cannot be excluded, and some relevant studies may have been missed. In addition, study selection was not conducted through independent duplicate screening, and the methodological quality or risk of bias of individual studies was not formally assessed. Accordingly, this review should be interpreted as a conceptual and integrative synthesis of the literature rather than an exhaustive or quantitative assessment of the available evidence.

## 7 Conclusion

Based on the literature synthesized in this review, we conceptualize CM as a translational approach at the intersection of clinical nutrition, precision nutrition, and medical practice. Rather than generating dietary prescriptions, CM renders them practicable, converting the targets of medical nutrition therapy, precision nutrition, and FIM programs into meals that patients can prepare and sustain. Its value lies not in promoting home cooking, but in aligning food preparation with clinical goals and real-world settings.

The mechanisms supporting this role are biologically plausible and mutually reinforcing, spanning culinary self-efficacy, the modification of biochemical exposure during preparation, and the combination of foods that shape nutrient bioavailability and postprandial responses. Because behavioral mediators necessarily precede clinical change, the current evidence shows the expected sequence, with behavioral and self-management outcomes improving more consistently than biometric endpoints. That improved culinary competence yields durable clinical benefit remains plausible though unproven, as adequately powered trials with extended follow-up are lacking.

Realizing the potential of CM requires that it be treated as an implementation strategy within nutrition care rather than a stand-alone cooking program. This demands explicit specification of intervention components and professional roles, design for the conditions under which patients actually live, and evaluation of both implementation and health outcomes. On these conditions, CM can serve as a rigorous bridge between nutritional science and everyday meal preparation, connecting clinical nutrition with medical practice.
